# Altered mRNA expression of genes related to nerve cell activity in the fracture callus of older rats: A randomized, controlled, microarray study

**DOI:** 10.1186/1471-2474-5-24

**Published:** 2004-08-03

**Authors:** Martha H Meyer, Wiguins Etienne, Ralph A Meyer

**Affiliations:** 1 Orthopaedic Research Laboratory, Department of Orthopaedic Surgery, Carolinas Medical Center, P.O. Box 32861, Charlotte, NC 28232-2861 USA

## Abstract

**Background:**

The time required for radiographic union following femoral fracture increases with age in both humans and rats for unknown reasons. Since abnormalities in fracture innervation will slow skeletal healing, we explored whether abnormal mRNA expression of genes related to nerve cell activity in the older rats was associated with the slowing of skeletal repair.

**Methods:**

Simple, transverse, mid-shaft, femoral fractures with intramedullary rod fixation were induced in anaesthetized female Sprague-Dawley rats at 6, 26, and 52 weeks of age. At 0, 0.4, 1, 2, 4, and 6 weeks after fracture, a bony segment, one-third the length of the femur, centered on the fracture site, including the external callus, cortical bone, and marrow elements, was harvested. cRNA was prepared and hybridized to 54 Affymetrix U34A microarrays (3/age/time point).

**Results:**

The mRNA levels of 62 genes related to neural function were affected by fracture. Of the total, 38 genes were altered by fracture to a similar extent at the three ages. In contrast, eight neural genes showed prolonged down-regulation in the older rats compared to the more rapid return to pre-fracture levels in younger rats. Seven genes were up-regulated by fracture more in the younger rats than in the older rats, while nine genes were up-regulated more in the older rats than in the younger.

**Conclusions:**

mRNA of 24 nerve-related genes responded differently to fracture in older rats compared to young rats. This differential expression may reflect altered cell function at the fracture site that may be causally related to the slowing of fracture healing with age or may be an effect of the delayed healing.

## Background

Bone formation to bridge the fracture gap following skeletal fracture slows with age in both humans [1-6] and rats [7-9]. While young, 6-week-old rats reach radiographic union by 4 weeks after femoral fracture, adult, 26-week-old rats require 10 weeks, and older, 52-week-old rats need in excess of 6 months [7]. Despite this increased time to radiographic union with age, there was no increase in the time of expression of Indian hedgehog or any of the bone morphogenetic proteins in the fracture callus for adult rats [10] or for older rats [11,12]. Radiographic union for adult and older rats occurred well after the time of expression of these skeletally active cytokines [10,11]. Except for markers of osteoblast activity and bone matrix formation, few genes remain up-regulated during the time period when bone forms to bridge the fracture gap [10-12].

These earlier studies done with RT-PCR revealed a paucity of data for genes differentially expressed by age. We had hypothesized that bone formation to bridge the fracture gap would be under a negative-feedback control system. Thus, the genes which stimulate bone formation should be up-regulated in adult or older rats to attempt to accelerate their slower progression of bony healing. This was not observed in adult [10] or older [11,12] rats. Either bone formation to bridge the fracture gap is not subject to negative-feedback control, or the genes up-regulated to control this bone formation are not those normally thought of as being involved in skeletal homeostasis. This suggested the need for a wider search for genes active during the fracture reparative process.

In this project, mRNA gene expression was measured by DNA microarray technology at various time points after fracture for young, adult, and older rats. The goal was to identify genes whose expression following fracture was altered by age. Such genes may either show reduced expression, if the age-related slowing of healing is caused by inadequate expression levels, or they may show enhanced expression, in an attempt to stimulate some poorly responding pathway. Among the genes which were differentially expressed at the fracture site with age were genes related to nerve cell activity.

In this study, we explored whether abnormal mRNA expression of genes related to nerve cell activity was associated with the slowing of skeletal repair in older rats. Abnormalities in the innervation of the fracture site will slow skeletal healing clinically [13-15] and experimentally [16-18].

## Methods

### Rats

Intact female Sprague-Dawley rats (Harlan Sprague-Dawley, Inc., Indianapolis, IN) were purchased at one or six months of age and housed in our vivarium in pairs until they were the proper age for experimentation. The rats were fed Teklad Rodent Diet [W] (#8604, Harlan Teklad, Madison, WI) and tap water *ad libitum*. The work was done in an AAALAC-accredited vivarium under protocols approved by our Institutional Animal Care and Use Committee.

### Surgery

Intact female Sprague-Dawley rats at 6 (young), 26 (adult) or 52 (older) weeks of age, weighing 154 ± 11 g (mean ± SD), 281 ± 25 g, and 330 ± 30 g respectively, were anaesthetized with an intraperitoneal injection of ketamine and xylazine (30 mg and 5 mg/kg body weight respectively) as described earlier [7,11]. The left knee was shaved, scrubbed with Betadine Solution (Purdue Frederick, Stamford, CT), and draped with sterile sheets. A medial incision was made at the knee, the patella was deflected laterally and a 1.0 mm hole was drilled into the intercondylar notch. An intramedullary rod (1.0 mm diameter, stainless steel, type 304V, O-SWGX-400, Small Parts, Miami Lakes, FL) was placed retrograde into the left femur [11]. The incision was closed with wound clips. A closed simple transverse mid-diaphyseal femoral fracture was induced with a Bonnarens and Einhorn device [19]. Randomly selected rats from among those scheduled for surgery were used for 0 time no-fracture sham controls. Rats were euthanized at 0, 0.4, 1, 2, 4, and 6 weeks after fracture for a total of 6 time points at each of the 3 ages. Six rats per time point per age group were selected for microarray analysis (2 rats/array). Radiographs were made at fracture, at 1 week after fracture, and at euthanasia. The femora were rapidly harvested, and one third of the femoral length, centered on the fracture site, was collected. This contained the fracture callus with associated cortical bone and marrow and was frozen in liquid nitrogen and stored at -75 C.

### RNA Sample Preparation and Microarray Processing

Samples were prepared as described in the Affymetrix GeneChip Expression Analysis Technical Manual (copyright 2001, Affymetrix, Inc., Santa Clara, CA, Rev. 1, Part number 701021, ). The sample preparation is described here in brief. Total RNA was extracted from the tissue by TRIzol (Invitrogen Life Technologies, Carlsbad, CA) with disruption of the tissue in a Brinkman Polytron homogenizer. RNA from two rats of the same age and time point was pooled for each microarray sample. Samples with 30 μg RNA were purified on RNeasy columns by Qiagen (Valencia, CA, P/N 74104) and then converted to double-stranded cDNA with a Superscript Double Stranded cDNA Synthesis Kit (Invitrogen Life Technologies, P/N 11917-010). The cDNA was then expressed as biotin-labeled cRNA by *in vitro *transcription (IVT) with the Enzo RNA Transcript Labeling Kit (Affymetrix, P/N 900182). Each sample was spiked with bioB, bioC, bioD, and cre (Affymetrix P/N 900299). The biotin-labeled cRNA was fragmented non-enzymatically. The fragmented cRNA was hybridized to 54 Rat U34A microarrays (Affymetrix P/N 900249) in the Affymetrix hybridization buffer for 16 hours at 45 C. The hybridized arrays were washed and stained in the Affymetrix Fluidics Station 400 to attach fluorescent labels to the biotin, followed by biotin-labeled antibody, and then a second staining with fluorescent labeling of the biotin. Each array was scanned twice by the Agilent GeneArray Scanner G2500A (Agilent Technologies, Palo Alto, CA). Three arrays from three independent samples (six rats) were done for each age at each time point.

### Data Analysis

The Rat U34A GeneChip Microarray has probe sets for over 8,700 rat genes. Most probe sets have 20 different probes for the same gene on each array with 20 additional mismatch controls. The data were analyzed with Affymetrix Microarray Suite 5.0 and Affymetrix Data Mining Tool 3.0 software. Microarray Suite was used to scale the mRNA expression (signal value) of all genes to an average of 500 for each array. For each gene, the software reported a signal value and a Present/Marginal/Absent call. This latter algorithm was a statistical comparison of the variation among the several probe sets for each gene compared to the noise level and gave a call for each gene as Present, Marginal, or Absent. The program then compared the signal value of each gene in the fractured samples against the signal value of the same gene in the unfractured control sample. The difference between the two signal levels, relative to the variability between the multiple probes for each gene, yielded a probability of change due to chance alone. Genes with p less than 0.005 were judged significantly different from the same gene in the unfractured sample. This more conservative p value was employed to minimize false positive responses.

The Data Mining Tool was used for cluster analysis with the Self Organizing Map (SOM) algorithm. The data were clustered on the signal values between 20 and 20,000 with the maximum/minimum ratio of at least 3.0 and the maximum – minimum difference of at least 100. One hundred clusters were specified.

Nerve-related genes were identified by searches for nerve-related names in the gene descriptions of each gene on the microarray. This association was confirmed by a review of the information for that gene in the NetAffx web site  and in the PubMed database . GenBank accession numbers and names are shown for each gene. Each graph shows the average ± SEM of the three microarrays that were done for each time point for each age. Significant changes in gene expression were demonstrated by t test and linear regression [20].

This report conforms to the MIAME standards of MGED . A copy of the full microarray data set has been deposited in the NCBI Gene Expression Omnibus  as series GSE594.

## Results

### Radiology

**Figure 1:**
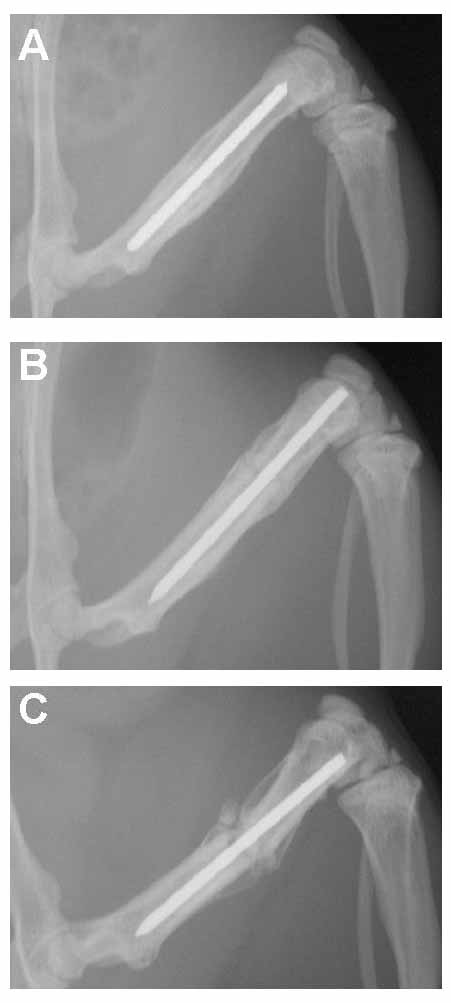
**Radiographs. **Radiographs of the fractured left femora at 6 weeks after fracture in rats 6(A), 26(B), and 52(C) weeks old at fracture.

In all young rats, bone bridged the fracture gap by four weeks after surgery. By six weeks after fracture, remodeling was beginning to obscure the fracture site (Fig. 1). In contrast, bone bridging in the adult rats progressed more slowly. The adult rats did have a vigorous periosteal reaction at the site of the fracture and were approaching radiographic union by six weeks after surgery (Fig. 1). In the older, one-year-old rats, bridging of the fracture gap by bone progressed the slowest. They had a minimal periosteal reaction at six weeks after surgery (Fig. 1).

### General results

On each array, on average, 5,200 genes were scored as absent, and 3,300 as present. Of these, 1,159 were significantly up-regulated and 928 were significantly down-regulated at two weeks after fracture in the adult rats of the first series (see Additional File 1). Up-regulated genes included cytokines and matrix genes for both cartilage and bone. Down-regulated genes included genes related to blood cell synthesis and mitochondrial function.

SOM clusters identified genes up- or down-regulated by fracture. Most genes affected by fracture followed the same time course at all three ages. These genes showed approximately the same peak expression level and regressed to baseline at about the same time point at all three ages. Among the genes affected by fracture were a number of genes associated with nerve cells. These were selected for more intense analysis.

### Similar responses at all three ages

**Figure 2:**
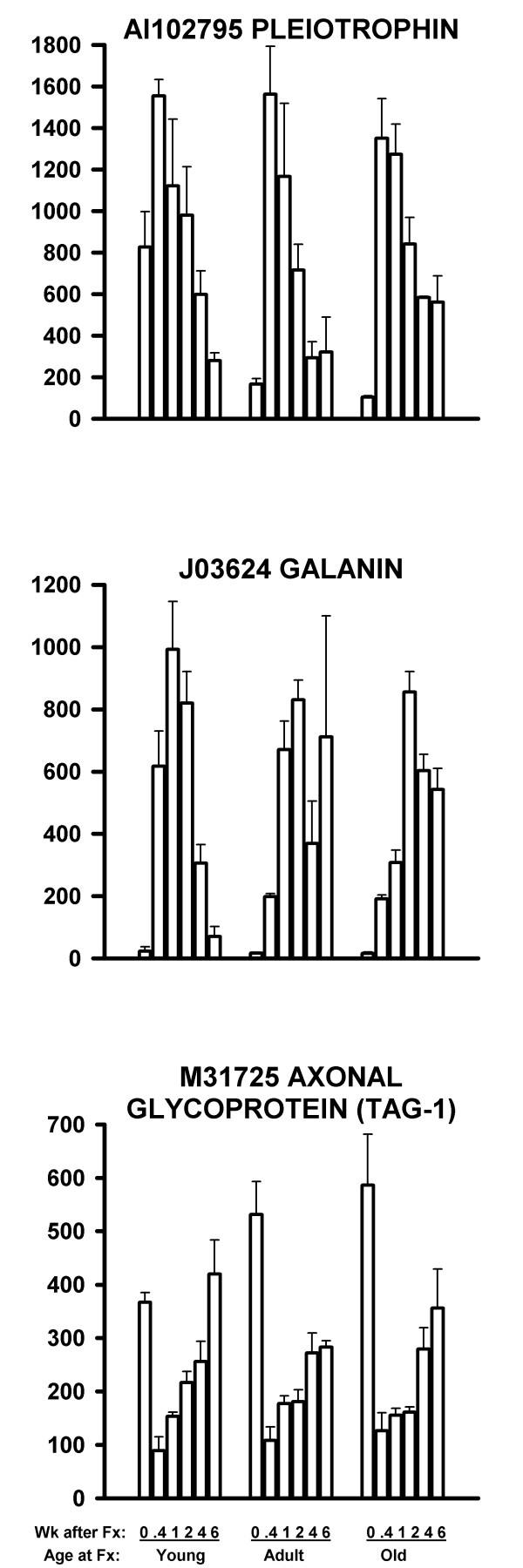
**mRNA levels of three nerve-related genes affected by fracture in young, adult, and older rats. **The first two genes were up-regulated at all three ages (0.4 week (top graph) and 2 weeks (middle graph) exceed 0 time control at P < 0.001), while the third gene was down-regulated at all three ages (0.4 week significantly less than 0 time control at P < 0.001). Rats were 6, 26 and 52 weeks of age at fracture respectively. Samples were collected at the indicated times after fracture. The 0 time samples were no-fracture controls. Each bar is the mRNA expression level for the indicated gene for the average ± SEM of three DNA microarrays in arbitrary units of fluorescence. mRNA from two rats of the same age and time after fracture were pooled for each array. Gene identifications are shown with their GenBank accession number. Axonal glycoprotein (TAG-1) is also known as contactin 2.

Up-regulated nerve-related genes are shown in Table 1. Two examples are shown in the upper two graphs in Figure 2. Both of these genes were significantly up-regulated from the 0 time control (P < 0.001 by t test for 9 samples (3 ages × 3 replicates) of 0 time *vs*. 0.4 week (Fig. 2, top graph) or *vs*. 0 time *vs*. 2 week (Fig. 2, middle graph)). Other nerve-related genes were down-regulated by fracture at all three ages (Table 2). These regained near normal activity by six weeks after fracture. An example is shown in the bottom graph of Figure 2. This gene (TAG-1) had a significant down-regulation after fracture (P < 0.001 by t test for 9 samples of 0 time *vs*. 0.4 week), followed by a significant increase at 6 weeks after fracture compared to 0.4 week after fracture (P < 0.001 by t test for 9 samples).

### Defects in the older rats

**Figure 3:**
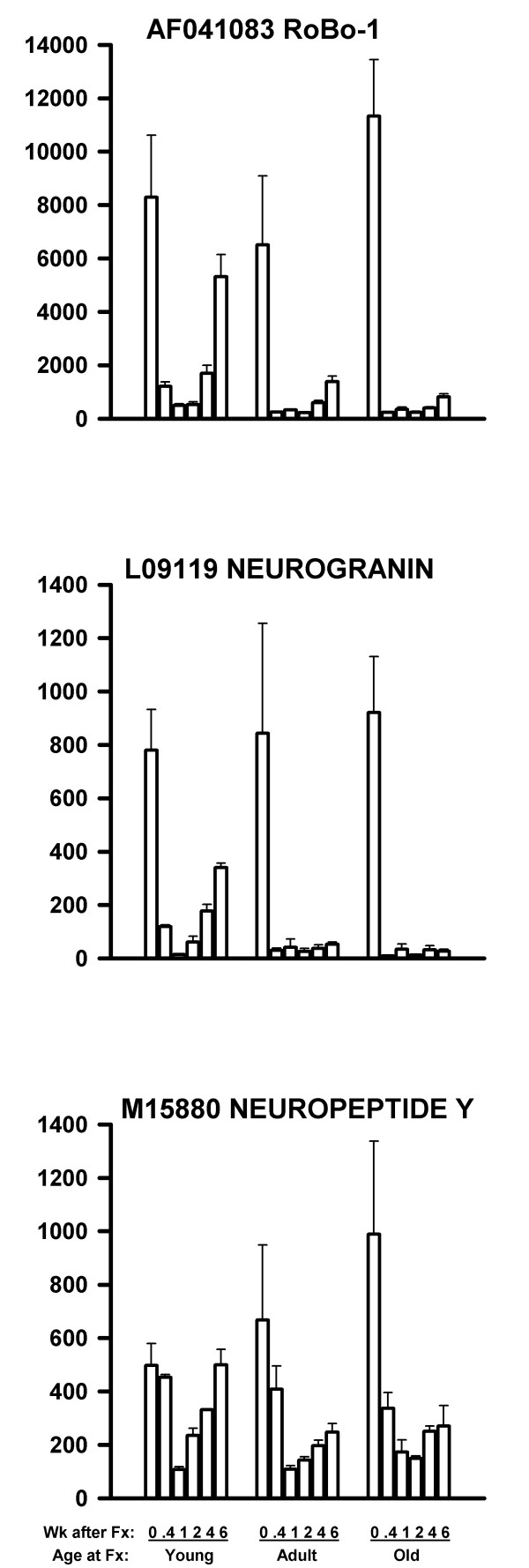
**mRNA levels of three nerve-related genes with more prolonged down-regulated after fracture in the older rats. **The 1 week values were significantly less than 0 time controls for all three graphs (P < 0.001). Note the failure of the mRNA activity to return to pre-fracture levels in the older rats (4 and 6 week values for young exceeded older rat values at P < 0.01). The data are presented as in Figure 2.

SOM cluster analysis identified three types of defects in the older rats. In the first type, a number of genes were down-regulated by fracture at all three ages. However, while genes in the younger rats were returning to pre-fracture expression levels by six weeks after fracture, there was less recovery in the older rats. These genes are shown in Table 3, and three examples of these genes are shown in Figure 3. All three of these genes had a significantly decreased mRNA expression levels at 1 week after fracture compared to 0 time control (P < 0.001 by t test for 9 samples (3 ages × 3 replicates)). At 4 and 6 weeks after fracture, the young rats showed faster recovery in mRNA expression than did the older rats for the three genes in Fig. 3 (P < 0.01 by t tests for 4 and 6 week young *vs*. 4 and 6 week old).

**Figure 4:**
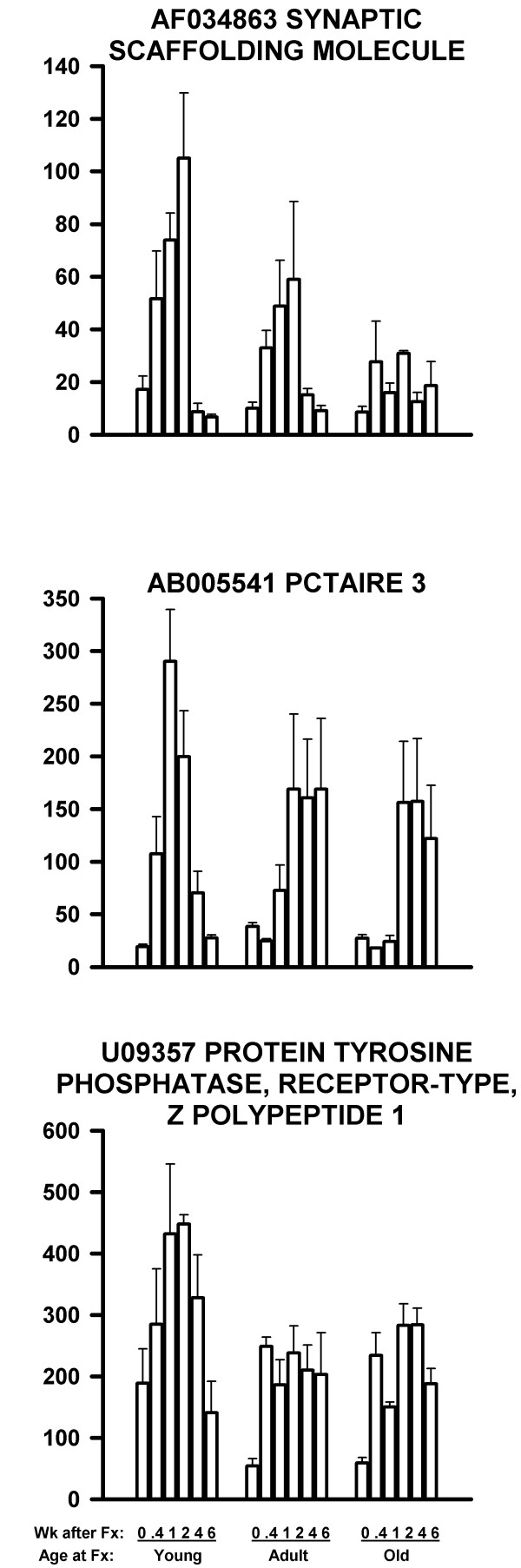
**mRNA levels of three genes related to nerve function and up-regulated more in young rats than in older rats following femoral fracture. **The decline with age was significant at P < 0.001 (top graph), P < 0.02 (middle graph) and P = 0.01 (bottom graph). The data are presented as in Figure 2.

In the second type of defect, other genes were up-regulated by fracture, but the response was weaker in the older rats. These genes are shown in Table 4. Three examples are shown in Figure 4. The broad peaks of the genes in Figure 4 permitted the t test to demonstrate a significantly higher expression level in the young rats at 1 and 2 weeks after fracture in comparison to the same time points of older rats. These comparisons for the three genes in Figure 4 were significant at P < 0.001 (top graph, AF034963), P < 0.02 (middle graph, AB005541) and P < 0.01 (bottom graph, U09357) for 6 samples per age group (2 time points pooled for 3 replicates).

**Figure 5:**
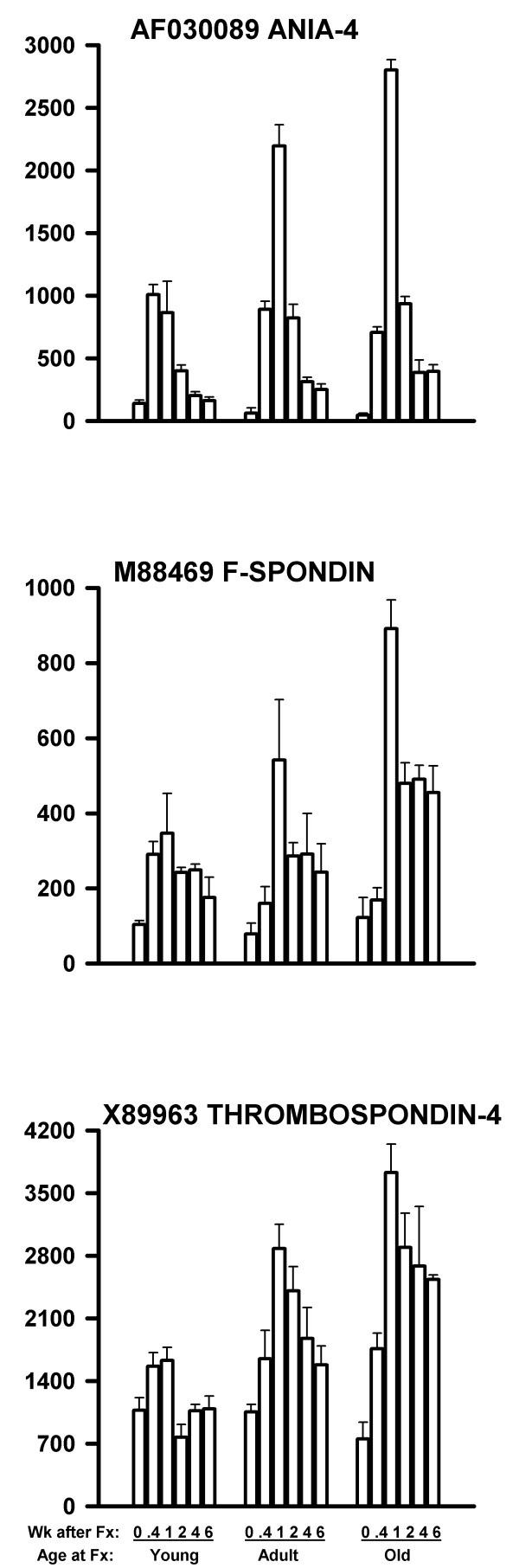
**mRNA levels of three genes related to nerve function and up-regulated more in older rats than in younger rats following femoral fracture. **The increase with age was significant at P < 0.001 (top graph), P = 0.01 (middle graph), and P < 0.001 (bottom graph). The data are presented as in Figure 2.

In the third type of defect, genes were also up-regulated by fracture. However, the response was stronger in the older rats than in the younger rats. These genes are shown in Table 5, and three examples are shown in Figure 5. The peak values for these three genes significantly increased with age by linear regression (P < 0.001 (top graph, AF030089), P = 0.01 (middle graph, M88469), and P < 0.001 (bottom graph, X89963) for 9 data points (3 ages × 3 replicates)).

### Present/Marginal/Absent calls

For each gene for each array, the Microarray Suite software reported a statistical decision as to whether the mRNA was Present, Marginal, or Absent. We have reviewed these calls for the genes shown in Figures 2,3,4,5. For Figure 2, the Present/Marginal/Absent calls were: Fig. 2 Top: 53/1/0; Fig. 2 Middle: 39/2/13; and Fig. 2 Bottom: 0/0/54. For Figure 3, the calls were: Fig. 3 Top: 45/2/7; Fig. 3 Middle: 7/0/47; and Fig. 3 Bottom: 32/6/16. For Figure 4, the calls were: Fig. 4 Top: 0/0/54; Fig. 4 Middle: 0/0/54; and Fig. 4 Bottom: 51/0/3. For Figure 5, the calls were: Fig. 5 Top: 45/1/8; Fig. 5 Middle: 52/0/2; and Fig. 5 Bottom: 54/0/0.

## Discussion

In this study, as in our earlier work [7,10-12], the time required to reach radiographic union after femoral fracture increased with age in the female rat. This slowing of fracture repair with age is associated with changes in the mRNA expression of specific genes within the healing fracture site [10-12]. To study this further, microarray technology was used to identify additional genes whose mRNA expression was affected by skeletal fracture.

More than two-thirds of the detectable genes on the rat U34A microarray have a change in mRNA expression level following fracture [21]. Most of these genes were not known to participate in the healing process of bone before the advent of microarray technology [21,22]. This reflects changes in both the types of cells at the fracture site as well as changes in the activity of the existing cells. Among the cells affected by fracture are nerve fibers. Protein and mRNA of genes related to neuronal functioning are found in intact bone and in the fracture callus [23-29]. Since proper innervation of the fracture site is needed for fracture repair clinically [13-15] and experimentally [16-18], this led to the hypothesis that the age-related slowing of fracture repair may be related to the abnormal nerve cell activity at the fracture site.

To evaluate this hypothesis, nerve-related genes were studied from among the genes present on the Affymetrix Rat U34A microarray. Genes were identified for which the mRNA response to femoral fracture was changed in the older rats compared to the young rats. Three types of change with age were found:

1. The mRNA expression levels of the genes shown in Table 3 and Figure 3 were decreased by fracture. While gene expression in the young rats was approaching pre-fracture levels by six weeks after fracture, gene expression showed minimal return to normal in older rats. Genes in this category were all related to signaling molecules or to signal receptors (references shown in Table 3).

2. Other nerve-related genes had strong up-regulation after fracture in young rats but only mild up-regulation in older rats. These are shown in Table 4 and Figure 4. This partial loss of function with age was observed in genes associated with nerve cell differentiation or cell cycle or genes related to synaptic structure (references cited in Table 4).

3. A third set of genes was increased in mRNA expression by fracture, but the increase was greater in the older rats. These are shown in Table 5 and Figure 5. Many of these genes were related to cell adhesion or to cell signal or signal transduction (references cited in Table 5).

All three classes of genes showed altered expression in the older rats compared to young rats. We hypothesize that bone fracture may physically disrupt nerve fibers in bone. A sub-population of these skeletal nerve fibers may regrow into the fracture site or regain function at a slower rate in older rats. This may account for the failure to recover from low mRNA values for the first group (prolonged down-regulation) or the failure to up-regulate mRNA expression adequately after fracture in the older rats in the second group (diminished up-regulation). Other genes in the third group with increased levels of mRNA after fracture in the older rats may represent attempts to stimulate nerve regrowth or other processes that are not responding. This may represent negative-feedback-induced up-regulation caused by effector cell resistance. Taken together, these changes in nerve cell function with age may contribute to the slowing of fracture repair in older rats. It must be pointed out that the associations noted here do not necessarily reflect cause and effect. It is also possible that the delayed re-innervation of the fracture site is an effect of the delayed healing in the older rats and not a cause of the delayed healing.

Experimental studies have been done to detect the role of innervation on fracture healing. Studies of sectioning the sciatic nerve in concert with tibial fracture have been reported to speed fracture healing [30-33]. However, sectioning both femoral and sciatic nerves inhibits fracture healing [18]. Aro *et al*. [16] have reported mechanoreceptors (Pacinian corpuscles) in the periostium of the rat fibula, which, if removed, lead to non-union [17]. Direct application of nerve growth factor to the fracture site increases healing in the rat rib [34].

In humans, abnormal bone healing is also associated with lack of nerve activity at the fracture site. Nagano *et al*. [13] have noted scaphoid nonunion in the wrists of patients with neuroarthropathy from a long-standing nerve palsy. Santavirta *et al*. [14] have found a lack of peripheral innervation at the fracture site of noninfected fractures with delayed union or nonunion of diaphyseal bones. Nordstrom *et al*. [15] have found a lack of stromal innervation associated with delayed union or pseudoarthrosis in spondylolysis.

Humans [1-6] show a slowing of fracture healing with increasing age as do rats [7-9]. The cause of the slowing of fracture healing with age is not well understood. The femora of young rats regain normal biomechanical properties by 4 weeks after fracture, while adults take 12 weeks, and older rats require in excess of 6 months [7]. This model presents an opportunity to elucidate novel genes important to this healing process. The slowing could reflect a loss of function as some processes essential for the rapid healing of fractures in young animals are inhibited with age. Alternatively, the slowing of skeletal repair with age may be caused by partial resistance of the healing process to stimulation in adult or older individuals. Such resistance should result in enhanced stimulation by regulatory systems to attempt to evoke a healing response. Both patterns were seen among the genes studied in this report. These genes are candidates for further study.

These changes with age are not limited to genes related to neuronal activity. We have also noted similar changes in genes related to mitochondrial activity [35]. It is likely that the age-related changes in fracture repair are caused by failure of several metabolic pathways. Methods, such as DNA microarrays, which sample many different biological pathways will be useful in defining these novel, multi-faceted defects.

The specificity of these changes is seen in the majority of the nerve-related genes for which the expression pattern following fracture was unaffected by age. These transcripts had similar increases or decreases following fracture in the young, adult, and older rats. These uniform responses suggest that most metabolic patterns were unaffected by age. Nerve-related genes similarly up-regulated by femoral fracture at all three ages were broadly related to differentiation and growth of nerve cells, to known up-regulation following nerve injury, or to association with apoptosis (references cited in Table 1). Some of these genes were slower to return to baseline values in older rats, such as galanin and TAG-1. In contrast, nerve-related genes similarly down-regulated by femoral fracture at all three ages were broadly related to the nerve growth cone or to synaptic signaling pathways (references cited in Table 2).

In this study gene expression was measured by quantification of the mRNA level for each gene with microarray technology. It must be kept in mind that there are other control systems which influence the protein synthetic rate and also protein degradation. Protein synthesis will be low in the absence of mRNA for that gene, but elevated mRNA levels are not a guarantee that protein levels will also be elevated for that gene. Changes noted at the mRNA level will need to be confirmed at the protein and structural levels. Assignment of the genes studied herein as nerve-related is made on the basis of currently available information. Other cell types in the fracture callus may also express these genes. Histological studies will permit the association of these genes with specific cell types within the fracture callus. These experiments are now in progress.

We have compared mRNA gene expression by microarray to that measured by reverse transcription – polymerase chain reaction (RT-PCR)[36]. Good correlation was found between the two methods if the transcripts were judged mostly present, the signal level did not approach the upper limit of the detector, and the probe sets or PCR primers were from the same region of the gene [36]. Some other genes, even though most samples were judged absent, also gave good correlation between the two methods. These latter genes were at the upper range of the absent calls and had good precision between samples [36]. The genes reported herein have the marked variation in mRNA levels that have been reported previously in fracture samples [10,11] with large changes in expression after fracture which return to the prefracture levels as healing progresses. The finding here of moderate signal levels, good precision among the three samples for each time point at each age, and a strong response to fracture indicate the ability of this technology to report changes in mRNA levels for these genes.

## Conclusions

In summary, most genes respond to bone fracture with altered mRNA gene expression, including genes related to neuronal functioning. However, a number of these genes responded to fracture differently in older rats than in young rats. Such differential expression with age may reflect altered cell functioning at the fracture site that may be related to the slowing of fracture healing in older rats.

## Competing interests

None declared.

## Authors' contributions

MM, WE, and RM participated in the surgery and the sample collection. MM and WE prepared and hybridized the samples and analyzed the data. RM prepared the manuscript. All authors read and approved the final manuscript.

## Pre-publication history

The pre-publication history for this paper can be accessed here:



## Supplementary Material

Additional File 1 Additional File 1. Response of adult rats at two weeks after fracture. This spreadsheet is a listing of all genes altered by fracture in the first adult rat sample at two weeks after fracture compared to the first adult no-fracture sample. The data are sorted on the probability value for the comparison of each gene between the two arrays. For each array, the signal (mRNA transcript level), detection (present, absent, or marginal) and detection p-value are given. In addition, for the two-week sample, the change (increase, marginal increase, marginal decrease or decrease) and change p-value are given for the comparison of the two-week sample to the no-fracture sample. These two arrays are archived in the GEO repository  as samples GSM9028 (no-fracture sample) and GSM9031 (2-week sample). Click here for file

## Figures and Tables

**Table 1:** Nerve-related genes up-regulated by fracture at all three ages

GenBank	Name	Reference
*Genes related to apoptosis*		
S67769	Cardiac sodium/calcium exchanger (Slc8a1)	[38]
M82824	Leucopus neurofibromatosis protein – type 1 (NF1)	[39]
L11319	Signal peptidase complex 18kD	[40]
*Genes up-regulated by injury*		
J03624	Galanin (shown in Fig. 2)	[41–43]
E12625	Rat novel protein expressed with nerve injury (sterol-C4-methyl oxidase-like)	[44]
*Genes related to nerve cell differentiation and growth*		
Z11558	Glia maturation factor beta	[45–47]
A03913	Glia-derived neurite-promoting factor (serine (or cysteine) proteinase inhibitor, clade E, member 2)	[48,49]
E05646	Hippocampal cholinergic neurostimulating peptide (phosphatidylethanolamine binding protein)	[50]
AB011531	MEGF 5, slit homolog 3	[51]
AA924772	Metallothionein 3	[52,53]
AF097593	N-cadherin, Testicular (cadherin 2)	[54,55]
AF023087	Nerve growth factor induced factor A (early growth response 1)	[56,57]
AI102795	Pleiotrophin (heparin binding growth factor 8, Hbnf, neurite growth promoting factor 1) (Fig. 2)	[58–60]
L19180	Receptor-linked protein tyrosine phosphatase type D (protein tyrosine phosphatase, receptor type D)	[61]
AF044910	Survival motor neuron	[62]
U64689	Synaptotagmin interacting protein zygin II (fasciculation and elongation protein zeta 2)	[63]
*Other genes*		
L07281	Carboxypeptidase E	[64,65]
U03416	Neuronal olfactomedin-related ER localized protein	[66]
U67140	PSD-95/SAP 90 associated protein-4 (disks large-associated protein 4)	[67]
M83680	RAB14 (GTPase Rab 14)	[68]
AF016247	RTK40 homolog (tyro 10, discordin domain receptor family, member 2)	[69]
X65454	SC65 – Synaptonemal complex protein	[70]
AJ006855	Synaptojanin 1	[71]
M96601	Taurine transporter (Slc6a6)	[72]
U35245	Vacuolar protein sorting homolog r-vps33b	[73]

Alternative names are shown in parentheses. References to physiological roles are shown to the right.

**Table 2:** Nerve-related genes down-regulated by fracture at all three ages

Genbank	Name	Reference
*Genes associated with the growth cone of nerve fibers*		
M31725	Axonal glycoprotein TAG-1 (Contactin 2) (shown in Fig. 2)	[74]
S82649	Neuronal activity-regulated pentraxin (Narp)	[75]
X59149	Neural cell adhesion molecule L1	[76–78]
*Genes associated with synaptic signaling pathways*		
L08496	GABA-A receptor delta subunit	[79]
S68944	Na^+^/Cl^-^-dependent neurotransmitter transporter	[80]
S76742	Neurotransmitter transporter rB21a (X transporter protein 3)	[81]
U39549	Synaptogyrin (synaptogyrin 1)	[82]
D28512	Synaptotagmin III (synaptotagmin 3)	[83]
AF007758	Synuclein 1 (synuclein alpha)	[84]
*Other genes*		
AF081557	Glial cells missing protein homolog (Gcm1, glial cells missing homolog a)	[85]
L15305	Glial-derived neurotrophic factor	[86]
M24852	Neuron-specific protein PEP-19 (Purkinje cell protein 4)	[87]
D17521	Protein kinase C-regulated chloride channel	[88]

Data presented as in Table 1.

**Table 3:** Nerve-related genes with prolonged down-regulation after fracture in older rats

GenBank	Name	Reference
L09119	C kinase substrate calmodulin-binding protein (neurogranin) (shown in Fig. 3)	[89]
S77867	G coupled protein receptor UHR-1 (G protein coupled receptor 10)	[90]
AF109405	GABA-B receptor 2 (G protein-coupled receptor 51)	[91]
U08290	Neuronatin alpha (neuronatin)	[92]
M15880	Neuropeptide Y (shown in Fig. 3)	[93,94]
S56508	Fatty acid coenzyme A ligase, long chain 6	[95]
AF041083	RoBo-1 (Slc11a1) (shown in Fig. 3)	[55,96,97]
X86789	Sensory neuron synuclein (gamma-synuclein)	[98,99]

Data presented as in Table 1. All genes are related to signals and signal receptors.

**Table 4:** Nerve-related genes with a greater up-regulation in younger rats

GenBank	Name	Reference
*Genes related to nerve cell differentiation and the cell cycle*		
X86003	Neuron-derived orphan receptor-2	[100]
AB005540	PCTAIRE 2	[101]
AB005541	PCTAIRE 3 (shown in Fig. 4)	[102]
U09357	Protein tyrosine phosphatase, receptor-type, Z polypeptide 1 (shown in Fig. 4)	[103]
*Genes related to the synapse and sensory perception*		
AI179632	Proton gated cation channel DRASIC	[104]
AF034863	Synaptic scaffolding molecule (S-SCAM, activin receptor interacting protein 1) (shown in Fig. 4)	[105,106]
S56141	Sodium-dependent neurotransmitter transporter	[107]

Data presented as in Table 1.

**Table 5:** Nerve-related genes with a greater up-regulation in older rats

GenBank	Name	Reference
*Genes related to signals and signal transduction*		
X74832	Acetylcholine receptor alpha subunit (cholinergic receptor, nicotinic, alpha polypeptide 1)	[108]
M16112	Brain type II calcium/calmodulin dependent protein kinase beta subunit	[109]
*Genes related to cell adhesion*		
M88469	F-spondin (shown in Fig. 5)	[110]
AF060879	Neurocan (chondroitin sulfate proteoglycan 3)	[111,112]
U16845	Neurotrimin	[113]
M96375	Non-processed neurexin 1-beta (neurexin 1)	[114]
X89963	TSP-4 (thrombospondin-4) (shown in Fig. 5)	[115–117]
*Other genes*		
AF030089	Activity and neurotransmitter-induced early gene 4 (ania-4) (shown in Fig. 5)	[118]
D88250	Serine protease (complement component 1, s subcomponent)	[119]

Data presented as in Table 1.
